# Report on the 56th ASH Annual Meeting, San Francisco, 4–9 December 2014

**DOI:** 10.3332/ecancer.2015.514

**Published:** 2015-02-26

**Authors:** Alberto Agazzi

**Affiliations:** Hematoncology Division, European Institute of Oncology, Milan, Italy

**Keywords:** chimeric antigen receptors, intensive chemotherapy, PD-1 inhibitors, target therapy

## Abstract

The 56th annual ASH (American Society of Haematology) meeting was held in San Francisco (CA). More than 3,000 abstracts were selected for presentation due to the huge amount of information from basic science to clinical experience. The future direction in haematoncology is targeted therapies for most diseases; for instance, anti-PD-1 and CAR-T cells in lymphoproliferative disorders and novel immunomodulatory agents active in the contest of bone marrow milieu in multiple myeloma. On the other hand, in aggressive haematological diseases (AML, ALL), clinical studies demonstrated the feasibility of a more intensive chemotherapy approach in older patients.

## AML in older adults

Acute myeloid leukaemia (AML) is a disease of older adults with a median age of 67 years at presentation. In the past, only one-third of older patients (> 65 years old) received definitive therapy due to concern about their overall fitness and potential treatment-related mortality. However, epidemiological data have shown that older patients with AML (up to 80 years old) have more tolerance and survival rate after therapy than their untreated counterparts [1]. Currently, therapeutic options for elderly patients include intensive chemotherapy with cytarabine and anthracycline backbone, hypomethylating agents (decitabine and azacitidine), low-dose cytarabine, investigational agents and supportive care with hydroxyurea and transfusions [2]. Preliminary results suggest benefit from reduced intensity allogeneic haematopoietic cell transplantation (HCT) in selected patients (60–80 years old) [3, 4]. In the absence of prospective randomized trials comparing HCT and chemotherapy, the decision to recommend HCT is based upon retrospective analysis of the risk of relapse and non-relapse mortality after each approach. It is strongly evidenced that pre-HCT comorbidities can predict HCT-related morbidity and mortality. Age alone does not appear predictive, and if the risk of relapse with chemotherapy is high, it should not be the sole basis for deciding against HCT. Disease status and pretreatment cytogenetics (FLT3-TID, NPM-1, CEPB-α) are the main factors predicting relapse, and these are likely to be supplemented by incorporating other molecular markers and the level of MRD after chemotherapy [5]. HLA-matched related and unrelated donors grafts seem to be better than those from other donor sources. Donor age is of no clear significance.

## AML (Intermediate risk therapy)

Research in molecular genetics has been crucial in deciphering the molecular heterogeneity of AML, in particular the subset of patients with intermediate risk cytogenetics [7]. At present, NPM1, CEBPA, and FLT3 have entered into clinical practice [8], although FLT3-inhibitors, including lestaurinib, sorafenib and midostaurin (phase 3 trials), crendanib, quizartinib (phase 2), are under investigation. The addition of immunoconjugate gentuzumab ozogamicin to standard induction therapy (7 + 3) has been shown to improve outcome [8, 9]. A common standard for post-remission therapy is repeated cycles of intermediate high-dose cytarabine. Allogeneic transplantation may offer a survival benefit for many patients in this setting. Many novel agents targeting mutant leukaemia drivers of deregulated pathways are in clinical development (DNMT3A, IDH1, IDH2, ASXL1, TET2, RUNX1).

## Myelodisplastic syndromes

Myelodisplastic syndromes (MDS) are the most commonly diagnosed myeloid disease with >15,000 new cases yearly in western countries [10].

Prognostic scoring systems divide patients into those with lower risk and those with higher risk MDS. Although treatment goals for patients with low-risk disease focus on minimizing transfusions and optimizing quality of life, the treatment goal for high-risk patients is to delay transformation into acute leukaemia and to prolong survival. In low-risk patients, cytopaenias are treated with erythropoiesis-stimulating agents or growth factors such as thrombopoietin mymetics [11]. For patients with del (5q-) or those who fail these initial approaches, lenalidomide may be tried, as can experimental agents [12]. Low-risk patients with multiple cytopaenias may be treated with immunosuppressive drugs or low-dose hypomethylating agents [13]. For patients with high-risk MDS, hypometthylating agents are the main initial treatment approach, but novel molecules are being developed for MDS patients who have failed when treated by hypomethylating drugs:

- Azacitidine (AZA) + Lenalidomide (rand. phase 2, N 280) OR 72% CR 44%.
- AZA + HistoneDeacetylase inhibitor (HDI) Vorinostat (rand. phase 2, N 280) OR 70%, CR 42%.
- AZA + HDI Pracinostat (rand. phase 2, N 100 ongoing; in phase 1 OR 89%, CR 78%).
- AZA + PI3K/PLK1 inhibitor Volasertib (phase 1 ongoing).
- PI3K/PLK1 inhibitor rigosertib (rand. phase 2 in hypomethylating agents failure. Median survival of 8.2 versus. 5.8 mo for control arm of best supportive care cytarabine).
- Decitabine + DNA methyltranserase depletion + anti-cancer quinolone-derivative vosaroxin (phase 1 and 2 ongoing. Of 16 patients, OR 81%).

## Double-hit lymphomas novel treatment strategy

Double-hit lymphomas (DHLs) are a heterogeneous group of mature B-cell lymphomas that harbour concurrent rearrangements of MYC and BCL-2 or occasionally BCL-6 [14]. They are associated with a very aggressive clinical behaviour and poor outcome after standard therapy (R-CHOP): OS less than 2 years [15]. It is also known that a significant proportion of diffuse large B-cell lymphomas (DLBCL) case have high coexpression of MYC and BCL-2 by IHC (double expressors): they are also associated with poor outcome after R-CHOP. Alternative therapies, (DA-EPOCH-R)and R-HyperCVAD, are under investigation and new approaches such as alternative immunotherapies and novel small molecules inhibitors (anti-MYC, anti-BCL-2 and BCL-6) [16].

## Chronic lymphocytic leukemia

Chronic lymphocytic leukemia (CLL) is characterized by a relatively small number of recurrent genetic alterations. Acquired defects in the p53 pathway, activating mutations of NOTCH1 and dysfunctional mutations of SF3B1 and BIRC3 identify patients with higher risk of progressive disease, poorer response to conventional therapies and shorter survival [17]. The patients with this CLL pattern should be candidates for a novel approach such as TKIs, BCL-2 inhibitors, monoclonal antibodies and immunological therapies including allogeneic transplantation and chimeric receptor-targeted T-cell.

## Follicular lymphoma and microenvironment

Immune and non-immune microenvironmental factors play a crucial role in the progression, transformation and resistance to therapy in follicular lymphoma (FL). We have recently experienced the development of several therapies targeting the microenvironment with activity in relapsed disease (immunomodulatory drugs, immune checkpoint inhibitors, immunoconjugates and small molecules inhibitors). These approaches are now at different stages of clinical development ranging from early trials in relapsed disease to phase 3 studies in an upfront setting now allow for the building of ‘chemo-free’ regimens.

Lenalidomide (phase 3 combination trials with monoclonal antibody and chemoimmunotherapy) is used as immunomodulatory drugs [18]. The immune checkpoint inhibitors used are ipilimumab (CTLA-4 inhibitor, phase I completed and combination trial considered) [19], pidilizumab, nivolumab, MK-3475 (PD-1 inhibitors, phase 2 single agent and combination trials) [20]. Brentuximab vedotin (anti-Cd30 Ab) is used as immunoconjugates, which includes phase-2 study. Ibrutinib (BRUTON’s TKI) is used as BCR pathway inhibitors, which include phase 2 and 3 including combinations [21].

## Multiple myeloma

Multiple myeloma (MM) is a unique cancer paradigm for investigating the mechanisms involved in the transition from a premalignant condition (MGUS) into a malignant disease (MM). The relationship among malignant plasma cells, the microenvironment and the genotypic characteristics of the MM clone plays a critical role in the outcome of the disease [22]. Although MM remains an incurable disease, the improved survival rates achieved over the past decade reflect the enhanced therapies (new generation IMIDS and proteasome inhibitors) for both upfront and relapsed disease. Moreover, new classes of agents are now in the development for MM [23]: oral proteasome inhibitors (Ixalomib and oprozomib), monoclonal antibodies (anti-CD 38 daratumumab and anti-CD138 Indatuximab), hystonedeacetylase inhibitors (panobinostat), AKT inhibitors (afuresertib), CDK inhibitors (dinaciclib) and immune therapies (PD-1 and PDL1).

## Conclusion

Immunotherapy was a big topic at ASH 2014 and novel therapies that harness the body’s own immune cells to attack cancer cells took centre stage. Anti-PD1 therapy was discussed extensively as a way of enabling the immune system to attach tumour cells. Also genetically engineered chimeric antigen receptor T (CAR T) cells were shown to be safe in patients with relapsed and treatment-resistant blood cancers. Genetics was also an important topic with presentations on sequencing circulating DNA as a diagnostic tool and looking for genetic mutations which may be drivers in promoting the disease. Again epigentics came to fruition this year and this was discussed extensively—the epigenetic state of cells within these malignancies will become more important. Finally, some new molecules, such as the monoclonal antibody daratumumab, appeared to work well with a variety of backbone regimens in multiple myeloma. Overall, in haematoncology, it is hoped that the new therapies will be entered into the clinic soon.
